# Factors determining the outcomes of immune imprinting after repeated orthoflavivirus infections

**DOI:** 10.3389/fimmu.2025.1560851

**Published:** 2025-07-16

**Authors:** Gielenny M. Salem, Fan-Chi Chen, James J. Cai, Day-Yu Chao

**Affiliations:** ^1^ Infection Biology Program, Global Center for Pathogen Research and Human Health, Lerner Research Institute, Cleveland Clinic, Cleveland, OH, United States; ^2^ Doctoral Program in Microbial Genomics, National Chung Hsing University and Academia Sinica, Taichung City, Taiwan; ^3^ Department of Veterinary Integrative Biosciences, Texas A&M University, College Station, TX, United States; ^4^ Graduate Institute of Microbiology and Public Health, College of Veterinary Medicine, National Chung Hsing University, Taichung City, Taiwan; ^5^ Department of Post-Baccalaureate Medicine, College of Medicine, National Chung Hsing University, Taichung City, Taiwan

**Keywords:** orthoflaviviruses, immune imprinting, original antigenic sin (OAS), antibody-dependent enhancement (ADE), antigenic seniority

## Abstract

Orthoflaviviruses, a group of arthropod-borne viruses, represent a significant global health threat, with hundreds of millions of infections each year, often leading to severe clinical outcomes. This Review elucidates the complexities of immune imprinting, also known as original antigenic sin (OAS), and its influence on immune responses to repeated, heterologous orthoflavivirus infections. We examine how initial exposure to a virus can shape subsequent immune responses, potentially resulting in sub-optimal binding of pre-existing antibodies to related but distinct viruses. Although OAS is often linked with adverse outcomes, such as enhanced disease severity in dengue due to antibody-dependent enhancement, we re-introduce the concept of “antigenic seniority,” which highlights the potential advantages of prior exposures by promoting cross-protection against related variants. This perspective underscores the dual nature of immune imprinting and its implications for vaccine development and therapeutic strategies against orthoflavivirus infections. By exploring the delicate balance between protective and maladaptive immune responses, we emphasize critical considerations for developing effective vaccines and interventions in the context of evolving viral threats.

## 1 Introduction

Orthoflaviviruses, primarily transmitted to humans by arthropod vectors, include several globally significant pathogens, such as Japanese encephalitis virus (JEV), Yellow fever virus (YFV), West Nile virus (WNV), dengue virus (DENV), and the newly emergent Zika virus (ZIKV) (1). These viruses are responsible for hundreds of millions of infections annually, often leading to severe clinical outcomes, such as encephalitis, hemorrhagic fever, and shock syndrome (2). Further, orthoflaviviruses are classified into serocomplexes based on antigenic relatedness, cross-reactivity, and cross-neutralization, including JEV (with WNV), DENV, Spondweni (with ZIKV), YFV, and tick-borne encephalitis virus (TBEV) (3). Antibodies against viruses within the same serocomplex can cross-neutralize related viruses but fail to do so across different serocomplexes, except in the case of DENV serotypes (4, 5). The co-circulation of multiple orthoflaviviruses in certain regions, coupled with increasing vaccination coverage for YFV and JEV and the modern surge in human geographic mobility, have heightened the likelihood of exposure to multiple orthoflaviviruses throughout a person’s lifetime. This raises the critical question of how pre-existing immunity, shaped by prior infections or vaccinations, influences the outcome of subsequent vaccination or infection with a heterologous orthoflavivirus.

**Figure 1:**
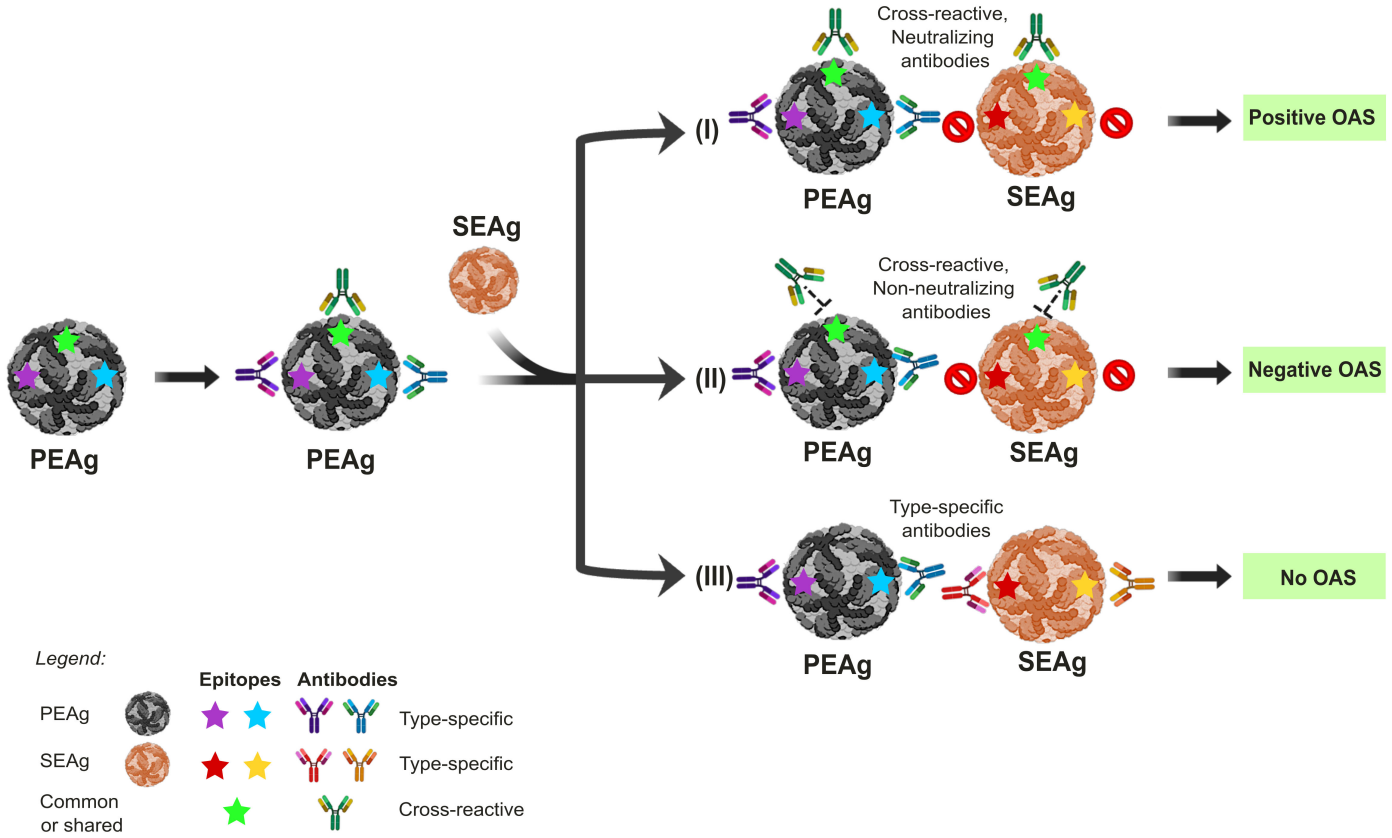
The impact of Original Antigenic Sin (OAS) on the outcomes of sequential orthoflavivirus infections. Exposure to the primary-encountered antigen (PEAg) or virus results in a long-lived memory B cell immune response, also generating type-specific antibodies that recognize epitopes labeled in blue or purple stars. Re-exposure to a secondary encountered antigen (SEAg), similar to PEAg, will result in three different scenarios of immune response outcomes, depending on whether the epitopes are conserved and the antibody is neutralizing. (I) Re-exposure to SEAg with the conserved epitopes labeled in green star induces a memory-boosting protective immune response to a cross-protective neutralizing antibody response. However, such a memory B cell response will prevent or significantly limit the ability of naïve B cells with epitope specificities to yellow and red stars from developing. In this scenario, although OAS results in a higher immune response to the PEAg than SEAg, the anti-Green antigen memory response confers some protection against SEAg infection, thus positive outcomes due to OAS. (II) In this scenario, the memory response to Green is non-protective, resulting in higher virus replication of SEAg through antibody-dependent enhancement (ADE), thus negative outcomes due to OAS. (III) This scenario depicts two separate exposures. Since no epitopes are shared between PEAg and SEAg, the antibody response to SEAg will be a primary exposure, unaffected positively or negatively by prior exposure to SEAg. The panels were original creations and selected source icons were created with Biorender.com.

When the immune system first encounters a foreign antigen (primary-encountered antigen, PEAg), such as a surface protein of a given virus, the immune response leads to the generation of specific antibodies and cytotoxic T lymphocytes. During the primary response, a fraction of the specific B- and T-lymphocytes will differentiate into memory cells. Memory B and T cells generated during primary immune response can rapidly respond to the same antigen upon re-exposure if they later encounter the same antigen. However, when the immune system encounters a secondary Ag (second-encountered antigen, SEAg), which is structurally similar but distinct antigen, the pre-existing antibodies induced from the PEAg bind to SEAg ineffectively (does not lead to virus neutralization), resulting in an impaired or ineffective immune response to the SEAg (**Figure 1**) (6–8). This phenomenon has been termed the original antigenic sin (OAS) and was first described by Thomas Francis Jr. in 1960 (9).

OAS has been implicated in several viral infections, including influenza, dengue, and SARS-CoV-2 (10–12). In the case of dengue, primary infection could induce cross-reactive antibodies but of non- or sub-neutralizing nature. These antibodies can enhance virus infection through Fcγ-receptor-mediated viral entry, known as antibody-dependent enhancement (ADE). ADE has been linked to more severe clinical outcomes such as dengue hemorrhagic fever (DHF) and dengue shock syndrome (DSS), with complications being more prevalent among patients with a history of DENV infection by the heterologous serotypes (11, 13).

While OAS generally implies the negative consequences of pre-existing immunity, it can be beneficial by offering protection against antigenically related virus strains (**Figure 1**). A more nuanced concept, “antigenic seniority,” provides a refined model of immune imprinting, highlighting how prior exposure to a pathogen shapes subsequent immune responses (12, 14). In this model, antibodies from initial exposure take a “senior position” in the immune framework, and responses to future infections or immunizations preferentially boost these pre-existing antibody responses while generating new, but often weaker, antibody responses. This back-boosting aspect of OAS can have a relative protective effect when novel virus variants emerge, such as has been shown for influenza or COVID-19 (15–17). For example, in the case of Omicron breakthrough infections among individuals vaccinated with the Wuhan (Wu) strain, immune responses were dominated by cross-reactive memory B cells (MBCs) targeting epitopes shared across multiple SARS-CoV-2 variants. This preferential recall may result from strong immune imprinting due to repeated exposure to the Wu S protein, which is antigenically dominant in bivalent vaccines (18). Exploiting this model of antigenic seniority will be key in developing vaccines with broader protection against SARS-CoV-2, SARS, MERS-CoV, and other viral infections (19). Although OAS or cross-reactive immunity has been extensively reviewed in DENV or ZIKV infections (20), a deeper understanding of antigenic seniority is essential for improving the effectiveness of vaccine development, particularly in the face of newly emerging orthoflaviviruses, and offers insights into why certain vaccines appear less effective against specific variants of concern.

The phenomenon of OAS exemplifies a double-edged sword of immunological memory: it can either enhance protection against closely related strains or hinder the immune system’s ability to elicit a protective response to novel viral variants. Since the geographic expansion of ZIKV circulation in Central and South America after 2016, there has been growing concern that prior immunity to DENV may exacerbate the severity of ZIKV infection (21). Although *in vitro* and *ex vivo* models suggested that the sera from individuals with prior DENV infection can enhance ZIKV infection, longitudinal cohort studies have not consistently shown that ADE leads to severe disease outcomes in ZIKV-infected individuals (22). The outcomes of an antibody response in protection or enhancement from infection are influenced by its magnitude, isotype, affinity, breadth, and duration, interacting with the heterogeneity of virion structures. Additionally, the effect of antibody feedback modulated by different immune cells *in vivo* has a positive or negative impact on the outcomes of viral infection, as recently reviewed (23), which may explain the discrepancies between the *in vitro* and clinical findings. The antigenic variability of the infected viral strains also plays an important role (24). Furthermore, a recent study suggests that prior exposure to JEV followed by DENV infection may induce broadly neutralizing antibodies capable of targeting not only JEV and DENV but also ZIKV, a virus the immune system has not yet encountered (25). In this Review, we examine the factors potentially influencing different clinical outcomes of immune imprinting, particularly focusing on the generation and selection of high-affinity B cells and how these processes could be either beneficial or harmful in the context of repeated orthoflavivirus infections or vaccination. While T-cell-mediated immune imprinting is also an important consideration, it will not be the focus of this Review.

## 2 Factors affecting B-cell-mediated immune imprinting

**Figure 2:**
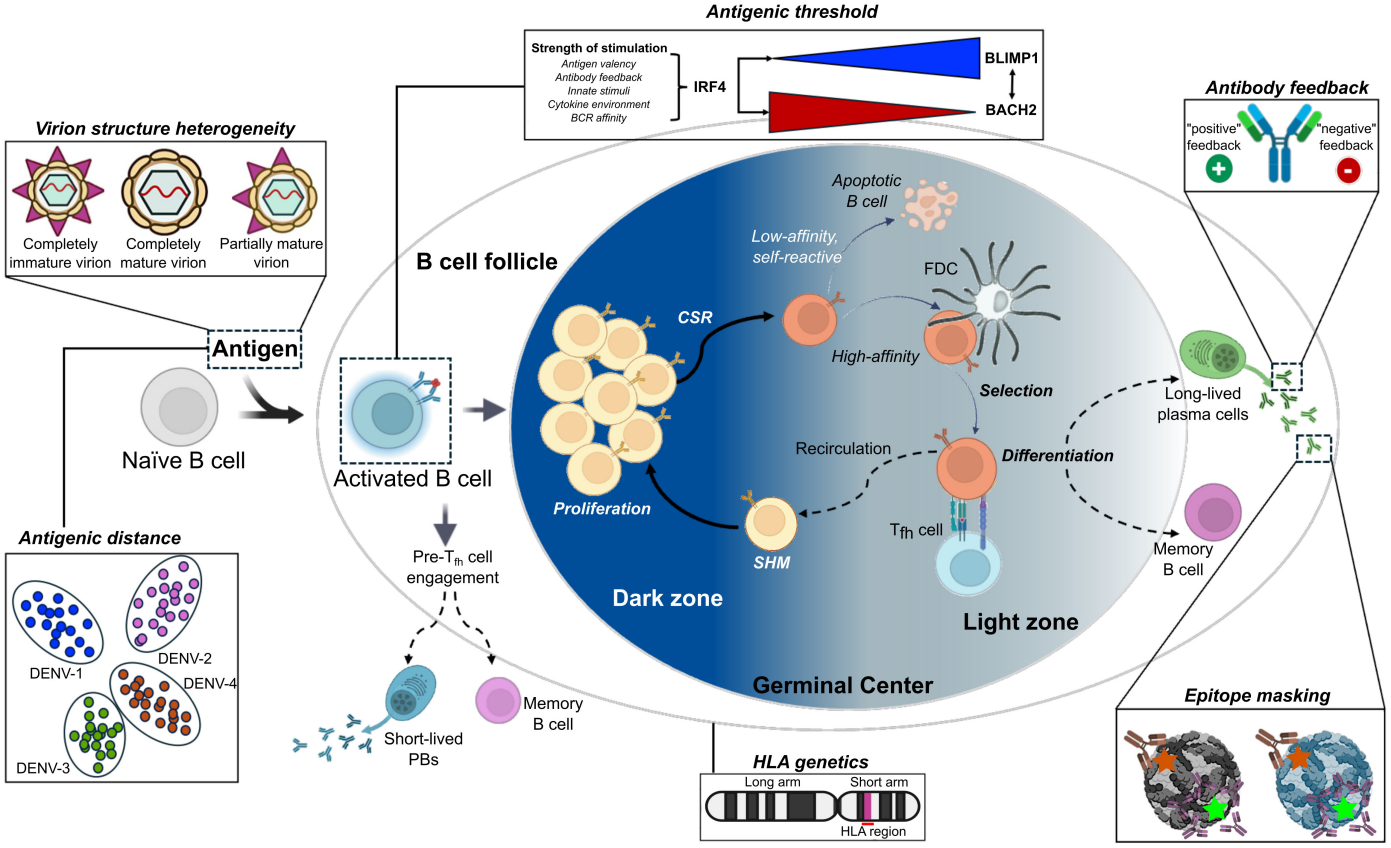
Germinal center dynamics and factors influencing the outcomes of an immune response upon repeated exposure to viral antigens. Upon initial antigen exposure, B cell responses to viral antigens generate plasmablasts (PBs) and long-lived plasma cells (LLPCs), which produce antibodies that neutralize, opsonize, and activate complement against specific antigens on the invading pathogen. Efficient interactions with the T cell receptor (TCR) and CD40 ligand (CD40L) expressed on antigen-specific follicular helper T cells (Tfh) determine the fates of activated B cells, which either lead to further B cell activation, proliferation and differentiation into long-lived PBs or into MBCs migrating to secondary lymphoid tissues for germinal center (GC) formations. MBCs are crucial components of long-term humoral immunity. Upon antigen re-exposure, memory B cells can rapidly become antibody-producing plasma cells or re-enter GCs to undergo further antibody somatic hypermutation (SHM), class-switch recombination (CSR), and affinity maturation. The re-participation of memory B cells in the GC reaction is important for generating broadly neutralizing antibodies against highly mutating viruses. Six factors influence the outcomes of an immune response upon repeated exposure to viral antigens, including antigenic distance, antigenic threshold, antibody feedback, virion heterogeneity, epitope masking from prior exposure, and host genetics. All panels were original creations with source icons created with Biorender.com. FDC, follicular dendritic cell.

Immune imprinting can lead to diverse B-cell-mediated immune outcomes upon repeated exposure to heterologous antigens. The nature of these outcomes is shaped by several key factors, which include (1) the kinetics of the immune response (i.e., the relative timing and magnitude of memory versus naïve B and T cell responses); (2) the affinity and functionality of antibodies generated; and (3) the breadth and diversity of the immune response at the time of antigen encounter. Additionally, the impacts of immune imprinting are strongly influenced by the degree of antigenic relatedness based on amino acid sequence composition (antigenic distance), the nature of the antigen (antigenic threshold), feedback of the antibodies induced from prior exposure, antigen conformation, glycosylation patterns, and any structural constraints imposed by epitope masking. The widespread distribution, shared antigenicity, and evolutionary relationships of orthoflaviviruses contribute to cross-reactive immune responses across multiple orthoflaviviruses (26, 27). Here, we will explore in greater detail how these factors dictate the nature and efficacy of the B cell response to repeated orthoflavivirus exposures (**Figure 2**).

### 2.1 Antigenic distance of prior exposures

The “antigenic distance hypothesis,” proposed by Smith et al., posits that the efficacy of vaccines is primarily influenced by the antigenic relatedness between previous vaccine strains, the current circulating variants, and newly emergent epidemic strains (28, 29). This framework offers insights into why individuals infected with H1N1 influenza viruses during childhood (and thus imprinted with H1N1-specific antigens) are subsequently protected against infections with antigenically similar strains, like H5N1, but exhibit less protection against more distantly related strains, such as H3N2. A parallel phenomenon can be observed with SARS-CoV-2, where exposure to the virus in late 2019 and its subsequent variants, either through infection or vaccination, led to significantly higher antibody titers against emerging variants, providing a degree of cross-protection (30).

In the context of orthoflaviviruses, within the *Flaviviridae* family, antigenic distance plays a crucial role in shaping immune responses. These enveloped viruses share a positive-sense single-stranded RNA genome that encodes 10 proteins, including the envelope (E) protein, which harbors key antigenic determinants (31). Although orthoflaviviruses are antigenically related, they exhibit genetic diversity in their E protein sequences. For instance, JEV and WNV share 79% sequence identity in their E proteins, whereas ZIKV displays an intermediate identity, ranging from 54–58% with JEV and WNV, and 40–46% identity with Yellow fever virus (32, 33). Antigenic distance, distinct from genetic distance, refers to how different the antigens (such as proteins on the surface of a virus or bacteria) of two different strains or variants are from each other. It is typically quantified by measuring the differences in epitopes between two strains or variants relative to the reciprocal neutralizing antibody titers. Despite the difference, the segregation observed in phylogenetic trees often mirrors, but not always, the division of orthoflaviviruses into distinct serocomplexes based on the extent of shared epitopes between strains (5, 27, 32, 34). A notable example is seen with dengue viruses, which cluster more closely based on the antigenic distance rather than their traditional serotype classification or sequence diversity (35). This has been demonstrated using antigenic maps constructed from neutralizing antibody titers in African green monkeys and human vaccination or infection data. These findings highlight how antigenic distance influences the degree of cross-reactivity, ultimately resulting in ADE or immune protection following exposure to different orthoflaviviruses. A recent study confirmed that the risk of hospitalization after dengue infection depends on the antigenic distance between an individual’s primary and secondary infections, with higher risk occurring at intermediate levels of antigenic distance (36, 37).

Although the negative imprinting effect of ADE through cross-reactive antibodies and its association with severe disease outcomes after repeated orthoflavivirus infection have been extensively reviewed (11, 13), the positive imprinting effect examples of cross-protection can be found in murine or human studies. In mice, prior exposure to orthoflaviviruses like DENV or YFV reduced viremia and mitigated the pathology during subsequent ZIKV infection, with the most robust protection being homologous primary ZIKV infection, followed by DENV-2 exposure (38). Further studies using immune-deficient AG129 mice highlighted that immunity to DENV-2 and ZIKV significantly suppressed YFV viremia, with humoral immune responses being the primary mediators of cross-protection, particularly after CD4+ or CD8+ T cell depletion (39). In humans, individuals in DENV-endemic regions who had repeated exposures to DENV exhibited higher neutralizing antibody (NAb) titers against ZIKV than those with a single DENV exposure (40), and no increase in ZIKV disease severity was observed in longitudinal studies conducted in Nicaragua (41–43). Conversely, prior ZIKV infection could enhance subsequent DENV infections, leading to more severe disease outcomes, a phenomenon that appears to be serotype-dependent (44, 45). Given that DENV and ZIKV share immunodominant epitopes and have similar antigenic distance, the differing outcomes depending on the order of infection may be affected by the structural differences between DENV 1–4 and ZIKV. These structural differences may modulate the virion thermostability, susceptibility to neutralization, and cell infectivity, as discussed in Section 2.3 “Antigen conformation”. Other potential explanations, such as the order of infection, discussed in Section 2.2.2, “Order of sequential antigenic exposure” could also play crucial roles in modulating host immune responses. Recent studies have identified specific cross-reactive human monoclonal antibodies that play a protective role against multiple orthoflavivirus from different sero-complexes. For example, antibodies recognizing the lateral ridge of domain III of the E protein in both ZIKV and DENV-1 have been shown to neutralize both viruses and protect mice from the ZIKV challenge (46). Similarly, cross-reactive antibodies between DENV and JEV recognize shared quaternary epitopes, leading to neutralization and protection against both viruses (25). In contrast, viruses such as tick-borne encephalitis virus (TBEV) and YFV are less antigenically related to other orthoflaviviruses, except for a shared fusion-loop epitope (FLE). Sequential exposure to TBEV and YFV, as seen in individuals pre-vaccinated with TBEV and later vaccinated with the YFV-17D vaccine, results in high cross-reactive IgG antibodies targeting the FLE. These antibodies exhibit poor neutralizing capacity but can enhance future DENV and ZIKV infections *in vitro*, highlighting the potential risks of immune imprinting in cross-reactive immune responses (47, 48).

Although there are debates between antigenic distance and the outcomes of immune imprinting, recent advances in molecular fate-mapping could further refine our understanding of how antigenic distance shapes immune memory. Schiepers and colleagues (49) introduced a novel fate-mapping approach that enables the tracking of the origins of the serum antibodies back to their specific B cell cohorts, providing insight into the dynamics of OAS. Their study demonstrated that sequential homologous exposures lead to a phenomenon termed “primary addiction”, where recall antibody responses are dominated by MBCs from the initial exposure, thereby suppressing *de novo* responses from naïve B cells. However, this imprinting effect sharply declined with increasing antigenic distance between priming and boosting antigens, allowing for the recruitment of new B cell clones. Although this work was conducted in the context of influenza and SARS-CoV-2, its principles and implications can be extended to orthoflaviviruses. The ability to quantify the relative contributions of primary versus *de novo* B cell responses holds promise for dissecting how antigenic distance between the primary and subsequent exposure antigens shapes the immune outcomes at the serum level, given the complexity of distinguishing pre-existing from newly activated B cell responses following heterologous infection or vaccination.

### 2.2 The nature of primary exposure

Upon initial antigen exposure, B cells in secondary lymphoid organs can function as antigen-presenting cells (APCs), which bind native, unprocessed antigens via their B cell receptors (BCRs), process them, and present the resulting peptides on major histocompatibility complex (MHC) class II molecules to CD4+ T cells (50). In contrast, when a B cell encounters an antigen that has been processed and presented on MHC class II molecules by another antigen-presenting cell (APC), such as a dendritic cell or macrophage, recognition of antigen-MHC complexes occurs indirectly through a helper T cell-mediated process. In this context, T helper cells (CD4+ T cells) engage with the MHC class II-bound antigenic peptides on the surface of the APCs via their T cell receptors (TCRs) and CD40 ligand (CD40L). This interaction facilitates B cell activation through cytokine signals and direct cell-to-cell contact, promoting a more robust immune response. This two-way interaction between B and T cells subsequently contributes to B cell activation, differentiation, and proliferation. These interactions ultimately determine whether activated B cells will differentiate into antibody-producing long-lived plasma cells (LLPCs) or MBCs, which migrate to secondary lymphoid tissues to establish GCs. MBCs are essential for long-term humoral immunity, providing rapid and enhanced responses upon antigen re-exposure. Upon reactivation, MBCs can rapidly differentiate into antibody-secreting LLPCs or re-enter GCs to undergo further rounds of somatic hypermutation and affinity maturation.

#### 2.2.1 Antigenic threshold

The antigenic threshold theory posits that the magnitude of BCR cross-linking alone does not solely dictate the quality and longevity of the humoral response. Instead, it suggests that a threshold of antigenic stimulation, achieved during infection or vaccination, is required to drive sufficient proliferation and differentiation of antigen-specific B cells into long-lived plasma cells capable of sustaining protective antibody titers over time (51). Vaccines containing multimeric antigens typically elicit robust BCR engagement in combination with CD4+ T cell help, thereby promoting the generation of durable LLPCs and conferring long-term immunity (51). Nonetheless, some multivalent vaccines provide only partial or transient protection despite presenting multiple epitopes, necessitating booster doses. These findings suggest that multivalency alone is insufficient to reliably determine the duration of protective humoral immunity. Rather, durable and long-term immunity depends on surpassing a critical antigenic threshold that ensures the generation of a stable pool of LLPCs necessary to maintain long-term serological memory.

MBC and LLPCs participation, particularly in the GC reaction, is thought to be crucial for generating and maintaining protective neutralizing antibodies, especially against rapidly mutating viruses such as HIV and influenza. Recent studies have begun to illuminate the mechanisms by which the fate of B cells is controlled, with transcriptional and epigenetic regulation emerging as central determinants of B cell differentiation (52). In particular, the IRF4-dependent imprinting of activation history has been shown to progressively shape B cell differentiation from naive cells to the terminal plasma cell stage. In this framework, if the stimulation is sufficiently strong to induce critical thresholds of IRF4 and BLIMP1 expression, B cells may bypass the GC and MBC stages and differentiate directly into LLPCs. This progressive differentiation model not only explains the gradual increase in LLPC output from GCs, as observed during COVID-19 infection, but also accounts for the variations in the quality and timing of the B cell response under different immunological conditions (12, 15). In severe cases of COVID-19, diminished Tfh cells impair the GC reaction, resulting in reduced affinity maturation and the emergence of MBC clones with low-affinity BCRs, indicative of impaired antibody responses. Similarly, immunization studies using domain III of West Nile virus (WNV) and Japanese encephalitis virus (JEV) have shown that pre-existing MBC diversity can limit the affinity maturation of the recall response, confining it to low-affinity clones; this suggested that MBCs in these cases were restricted by pre-existing clonal diversity, leading to the selection of low-affinity clones (53).

A similar phenomenon has been observed with the tetravalent dengue vaccine, Dengvaxia^®^, which included the structural proteins from four DENV serotypes (DENV1-4) on a YFV-17D backbone. Despite its multivalent design, the vaccine failed to confer sufficient efficacy in dengue-naïve individuals and was effective primarily among those with prior exposure to dengue viral infection (54). In contrast, other vaccines, such as the JEV vaccine, have been shown to induce more durable immune responses. The chimeric nature of Dengvaxia^®^ vaccine induces a different immunological profile compared to other orthoflavivirus vaccines containing single viral entity, such as the JEV vaccine strain SA14-14-2. Although the underlying mechanism remains to be explored, it is likely that strong protection can be achieved if a single vaccine dose is sufficient to induce critical thresholds of IRF4 and BLIMP1 expression (55). Dengvaxia^®^ targets four DENV serotypes, likely requiring a more complex immune response tailored to each serotype’s epitopes, as shown in the influenza vaccine (56). Its efficacy varies with prior dengue exposure, such that individuals with prior infection benefit from enhanced protection, whereas seronegative individuals may face an increased risk of severe disease upon subsequent infection (57). This underscores the importance of the antigenic threshold and the importance of eliciting appropriate Tfh cell responses. Investigating how immune imprinting influences vaccination outcomes, especially in tetravalent vaccines, will provide valuable insights into immunity and vaccine development (58).

#### 2.2.2 Order of sequential antigenic exposure

The order in which antigenic exposures occur plays a critical role in shaping the immune response, particularly during infections with closely related viruses. This principle, well-illustrated during the COVID-19 pandemic, revealed that sequential exposures to antigenically distinct variants of SARS-CoV-2 may blunt the development of robust immune “memory” (59). Similar findings have been exemplified by studies in primates and humans involving sequential orthoflavivirus infections. In DENV infections, heterologous secondary infections, distinct from the primary exposure, are associated with increased disease severity through ADE. Yet, recent studies suggest that this risk of hospitalization in secondary DENV infections is not solely determined by the serotype combination but also by the specific order of infections and the antigenic distance between the primary and secondary strains (36, 37). Experimental studies in non-human primates (prior exposure to DENV-3 or ZIKV, followed by secondary ZIKV infection) further support this concept. In rhesus macaques, hallmarks of immune imprinting were supported by (i) altered plasmablast response, (ii) reduced BRC diversity and somatic hypermutation, (iii) boosting of pre-existing DENV-3 neutralizing antibodies, and (iv) non-reciprocal cross-reactive IgG. Firstly, prior DENV-3 exposure led to a higher proportion of IgG-expressing plasmablasts during subsequent ZIKV infection, indicating isotype skewing shaped by DENV-3 priming. Single-cell sequencing, which tracked clonal expansion, revealed that DENV-3 primed animals also displayed lower somatic hypermutation and reduced variable gene diversity, reflecting a constrained, imprinted B cell response. ZIKV infection also triggered a 2- to 3-fold increase in pre-existing DENV-3 neutralizing antibodies, consistent with an anamnestic response. Lastly, DENV-3 infection elicited persistent ZIKV-binding IgG, but not the reverse, highlighting how infection order shapes immune memory (60). Another study on non-human primates with a tertiary DENV-4 infection showed that the order of prior exposure to DENV-2 or ZIKV influenced the immune response in early control of viremia, even in the absence of CD4+ T cells (61). Additionally, high levels of broadly cross‐reactive antibodies were found in samples from TBEV-infected patients pre-vaccinated with the YFV vaccine as well as in DENV patients pre-vaccinated against TBEV and/or YFV vaccines. While the cross‐reactive antibodies from YFV vaccination did not neutralize TBEV, they were effective in neutralizing DENV and dengue virus infections (62). Similarly, prior ZIKV infection enhanced subsequent DENV disease severity, but not the reverse order (44, 45).

The possible mechanistic explanation for these order-dependent outcomes is the effect of antibody feedback, a regulatory process first documented in 1909 (63). Antibody feedback can either enhance or suppress humoral responses by modulating antigen availability and B cell selection. As discussed by Cyster and Wilson (23), this mechanism exerts both stimulatory and suppressive effects on GC dynamics, modulating B cell selection, affinity maturation, and clonal expansion. In the context of sequential orthoflavivirus infections, pre-existing antibodies from prior exposure can limit antigen accessibility through epitope masking, thereby dampening the recruitment of naïve B cells and favoring the reactivation of MBCs targeting conserved, often subdominant, epitopes. A well-documented form of negative antibody feedback is antibody-dependent enhancement (ADE), where non-neutralizing or sub-neutralizing antibodies facilitate viral entry into Fcγ-receptor-bearing cells, exacerbating viral replication and contributing to severe disease. In dengue, ADE has been mechanistically and epidemiologically linked to severe outcomes during secondary infections with heterologous serotypes (11, 34). However, antibody feedback is not solely detrimental. In early phases following primary infection, cross-reactive antibodies may transiently provide heterotypic protection, particularly if neutralizing titers remain high (64). Beyond the acute phase, though, immune imprinting by previous orthoflavivirus exposures can bias MBC responses toward previously encountered epitopes. Notably, upon secondary exposure, MBCs preferentially re-engage familiar epitopes; sometimes at the expense of generating new, virus-specific neutralizing responses (65). Moreover, circulating antibodies may enhance antigen uptake though immune complex formation, promoting presentation and germinal center activity, and potentially boosting responses to shared antigenic sites. These contrasting forces of antibody feedback are relevant in shaping the breadth and quality of MBCs responses during heterologous infections, directly influencing outcomes such as cross-protection or ADE.

### 2.3 Antigen conformation

Antigen conformation refers to the structural characteristics of the antigen. The structural characteristics of the epitopes and their spatial arrangement are crucial in preferential recognition by the host to elicit humoral response and are influenced by the (i) heterogeneity of virions, (ii) their dynamic conformational changes, and (iii) immunodominant epitopes.

#### 2.3.1 Heterogeneity of orthoflaviviruses

The heterogeneity in orthoflavivirus antigenic structures is dictated by variable mature, partially mature, or immature virion status due to differences in the maturation process (66). The maturation process of orthoflaviviruses is a key determinant of the structural diversity, resulting in heterogeneous virion particles, and may explain the complexity of disease outcomes. During replication, orthoflaviviruses undergo a series of conformational changes, forming non-infectious virions with icosahedral symmetry composed of prM and envelope (E) protein heterotrimers (67). As these particles transit through the acidic environment of the Golgi apparatus, prM proteins are cleaved by the cellular protease furin, leading to rearrangement of E proteins into a herringbone pattern. This maturation process results in a smoother and infectious virion, while the incomplete prM cleavage yields partially mature, mosaic virions that exhibit mature and immature structural features (68–70). These heterogeneous and dynamic infectious particles circulate in infected hosts and may interact at various sites at the host cell surfaces and the immune system, which adds complexity to the immune responses, as antibodies may differentially recognize mature, immature, and mosaic virions (68, 71, 72). In DENV infections, such heterogeneity of virion particles has been suggested to be influenced by serotypes, genotypes, or strains, viral passage history, and the target cell types supporting viral replication (67, 68, 73). The variation in the virion population, coupled with antigenic differences between virus strains, could impact the immune response and, by extension, the disease severity (22, 44). Additionally, studies showed that the compositions of the polyclonal sera after repeated exposures to heterogeneous orthoflaviviruses were highly variable (5, 74). Since the traditional neutralization assays cannot fully capture the protective potential of the polyclonal response, a recent study suggests that using mature viruses could serve as correlates of protection (75).

#### 2.3.2 Dynamic conformational changes of the E glycoprotein

Besides heterogeneity of orthoflavivirus particles, extensive studies observed the temperature-dependent large-scale morphological changes, termed “virus breathing,” that expand the envelope (E) glycoprotein structure and affect the exposure of antigenic sites (76). The dynamic conformational changes of E proteins during maturation are also crucial for virus-cell membrane fusion (72). The E protein is structured into three domains: I, II, and III (33, 72). A critical domain within domain II is the fusion loop (FL), which is highly conserved among all orthoflaviviruses and pivotal in viral fusion and host cell entry (77). During viral maturation, the hydrophobic residues of FL are buried in the adjacent E monomer, making them less accessible on the mature virions unless the particles undergo conformational changes, such as those occurring during a viral expansion (i.e., during fever, where the virus “breathes” or in a partially mature stage). However, upon viral entry, conformational changes induced by the acidic pH in endosomes allow the FL to be exposed, and consequently generation of antibodies that recognize and bind to this epitope (78). Notably, these small-scale protein dynamics consequently influence their interactions with the immune system and infectivity.

#### 2.3.3 Immunodominance

Certain epitopes on the virus tend to elicit a stronger immune response over others. This phenomenon, referred to as immunodominance, occurs because the immune system more efficiently recognizes these epitopes due to their accessibility or higher affinity for peptide-MHC interactions (79). The E protein is the major target of neutralizing antibodies and constitutes epitopes that vary in immunogenicity, such as immunodominant, subdominant, or rare epitopes. Immunodominant epitopes are the primary antigenic peptides that preferentially initiate most of the immune response during initial antigen exposure (80). Among orthoflaviviruses, the fusion loop (FL) often serves as an immunodominant epitope, frequently targeted by both primary and secondary infections (34, 81). Antibodies targeting the FL region of E protein are further categorized into FLE- (FL-E monomer) and EDE-(envelope dimer epitope) recognizing antibodies (71). Given the conservation of the FL peptide, these FLE-directed antibodies dominate the immune response during orthoflavivirus infection, are generally cross-reactive across all orthoflaviviruses, but are often weakly neutralizing (34). Evidences showed that FLE antibodies contributed to ADE-associated severity upon dengue secondary infection (82, 83). A cluster of antigenic determinants at the tip of the TBEV E protein FL peptide revealed that these determinants are cryptic and mostly inaccessible at the surfaces of infectious virions, explaining the lack of efficient neutralizing activity (77). This immunodominant targeting of the FL likely reflects both its structural conservation and its recurrent presentation during infection, which can skew the antibody repertoire and affect subsequent responses through immune imprinting. Thus, these FL-directed antibodies can outcompete responses to more protective, subdominant epitopes such as the EDE. Broadly neutralizing EDE antibodies recognize the interface between two E monomers (71, 84), and target the mature and intact virion, neutralizing all four serotypes of DENV and ZIKV, thereby offering broader protection (34, 85). Though highly neutralizing, antibodies targeting domain III are typically less prevalent and constitute only a fraction of the humoral response (86). A recent study also showed that using different forms of the E protein (monomeric and dimeric) for absorption assays to dissect the complexity of polyclonal sera after repeated orthoflavivirus exposures is critical in determining actual protective capacity against subsequent infections (48). Finally, rare or non-immunodominant epitopes are seldom targeted by the immune system but may become more prominent by repeated exposures or in certain individuals, as in the case of potently cross-neutralizing EDE antibodies or antibodies recognizing the C-C’ loop of domain III (87, 88). These non-immunodominant but functionally relevant antibodies may gain therapeutic advantage against orthoflavivirus infections (89). Overall, this skewed epitope targeting leads to the preferential recall of immunodominant epitopes and shape the humoral response, even when they are not the most effective at neutralization.

Additionally, immunodominance is influenced by the intrinsic affinity of peptides for MHC molecules. Studies showed that peptides with higher affinity for MHC class II molecules are more likely to be presented and recognized by CD4+ T cells, leading to a dominant immune response against those epitopes (79). The relative positioning of B and T cell epitopes within an antigen can also influence immunodominance (90). Regions bound by immunodominant antibodies are often adjacent to CD4 epitopes, potentially boosting their presentation and recognition by T cells (90). For example, DENV-specific CD4+ T cell recognized epitopes within the E protein, particularly in E domain III and the E-dimer region (91).

### 2.4 Epitope masking by cross-reactive antibody or glycan composition

The concept of epitope masking postulates that pre-existing antibodies, directed against conserved but non-neutralizing epitopes, promote antigen clearance while simultaneously inhibiting the ability of novel antigens to engage with memory and naïve B cells. This mechanism restricts *de novo* antibody production against similar or cross-reactive epitopes (92). In the case of heterosubtypic infections involving orthoflaviviruses, such as those caused by DENV serotypes or ZIKV, plasmablast proliferation is triggered, generating cross-reactive antibodies that preferentially target epitopes associated with prior orthoflavivirus infections, as observed in both human and animal models. However, these antibodies may also lead to suboptimal immune responses by binding weakly to the boosting antigen. This occurs through multiple low-affinity interactions with the surface B cell receptor, expressed at high density on MBCs. As a result, while the immune system generates substantial quantities of the soluble antibody, the resulting antibodies may not effectively neutralize the virus.

**Figure 3:**
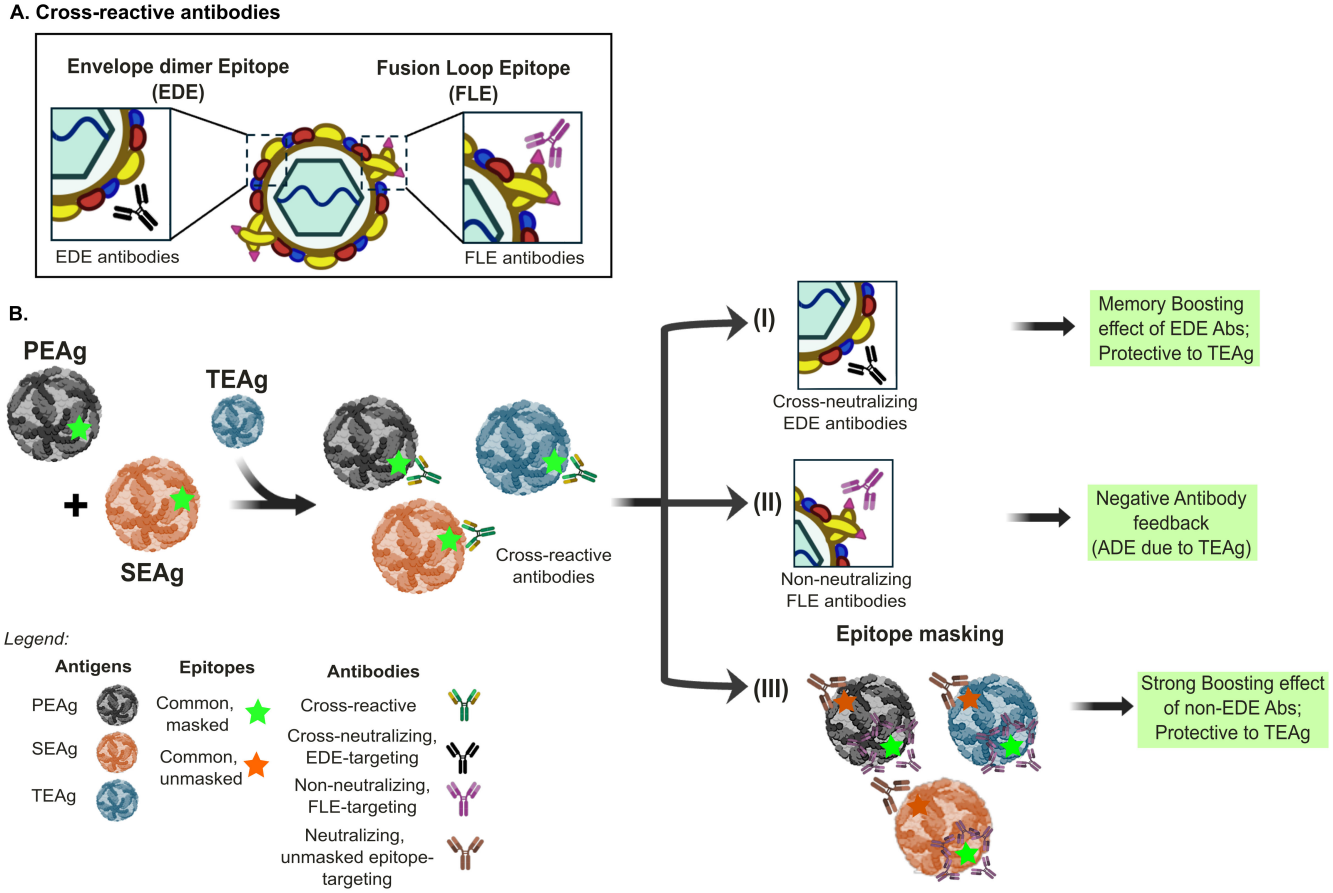
Epitope masking and Immune response outcomes in sequential Orthoflavivirus infections. There are three different scenarios of immune response outcomes to the tertiary encountered antigens (TEAg) after sequential exposure to primary encountered antigen or virus (PEAg) and secondary encountered (SEAg) in orthoflavivirus infection. **(A)** The cross-reactive antibody response after orthoflavivirus infection can be divided into envelope dimer epitope (EDE)-recognizing and fusion loop E-monomer (FLE)-recognizing antibodies. **(B)** (I) Re-exposure to SEAg with the conserved epitopes, labeled in green star, to all three antigens can induce a memory-boosting protective immune response to cross-reactive neutralizing EDE-like antibody response. (II) In contrast, such conserved and immunodominant epitope to TEAg, such as the FLE, could result in higher virus replication of TEAg through antibody-dependent enhancement (ADE). Hence, the memory response to the Green epitope results in negative antibody feedback and is nonprotective. (III) This scenario depicts that the memory response to the Green epitope can mask the FLE epitope, which is usually immune dominant. Consequently, the memory response to other conserved non-immune dominant epitopes labeled in orange could be boosted, providing a protective effect to TEAg. The panels were original creations and selected source icons were created with Biorender.com.

An alternative mechanism proposed by the epitope masking theory suggests that pre-existing antibodies that bind conserved epitopes can occlude immunodominant regions of the antigen (specifically the FLE epitopes), thus preventing them from being recognized by new B cells. This masking effect may paradoxically promote the formation of a *de novo* B cell response that targets the unmasked epitopes, which are rare or hidden but conserved across different orthoflaviviruses, such as those targeted by EDE antibodies, that were not encountered during the primary infection (**Figure 3**). A recent study demonstrated that glycan masking can be harnessed to selectively display epitope for antibody discovery (93). By introducing engineered N-glycosylation sites into DENV-2 EDIII, Nilchan and colleagues effectively shielded other known epitopes and only displayed the target binding sites. This strategy enabled the identification of highly potent neutralizing antibodies while minimizing ADE effects. Similarly, applying glycan masking to the N-terminal domain (NTD) and receptor-binding domain (RBD) of SARS-CoV-2 vaccine strain refocused the B cell responses onto the desired neutralizing epitopes, without compromising the antigen’s overall folded structure (94). These approaches offer a promising strategy for developing vaccines and therapeutics with a more targeted immune response and reduced risk of disease enhancement.

Orthoflaviviruses undergo post-translational modification with N-linked glycan chains, which are crucial for protein folding, stability, and virus entry. The glycosylation patterns on the virion surface, mainly on the E protein, can affect virion production (95, 96), susceptibility to neutralization (97), pathogenicity (98), and ultimately, modulate the immune response (22). In particular, contemporary ZIKV strains, associated with large outbreaks and neurodevelopmental malformations, possess an N-linked glycosylation site at position 154 (N154) on the E protein, which is absent in many historical strains (98). These glycosylated ZIKV strains demonstrated increased pathogenicity in *ifnar^-/-^* mice following peripheral administration compared to nonglycosylated variants. This difference may be attributed to enhanced attachment and infection of DC-SIGN- or lectin-expressing cells (98). The type of glycan, the differences in glycan composition among orthoflaviviruses, and the specific arrangements of these carbohydrate moieties on the virion surface can mask or expose epitopes, influencing neutralization potential by antibody recognition, and their ability to be recognized during sequential infections (97).

DENV contains two N-linked glycosylation sites at positions 67 (N67) and 153 (N153). While N153 (or N154 in ZIKV, WNV, and JEV) is conserved among orthoflaviviruses, N67 is unique to DENV and has been shown to enhance virus replication and tropism towards dendritic cells and macrophage-derived cells (22, 99, 100). This interaction was highly facilitated by high-mannose glycans, whereas complex sugars strengthen binding to DC-SIGNR. JEV mutants with a single or extra glycosylation at N67 increased binding to DC-SIGN, while wild-type JEV, with a single glycosylation site at N154, showed effective replication and a neurotropic advantage (101). Additionally, EDE-targeting neutralizing antibodies (EDE1 and EDE2) behave distinctly with ZIKV and DENV-2 due to the N67 glycan. EDE1 is glycan-independent and effectively neutralizes both viruses, whereas EDE2 requires N67 for neutralizing DENV-2 but enhances ZIKV infection (102, 103).

Besides the E protein, the prM protein is also glycosylated in various orthoflaviviruses. In DENV, glycosylation occurs at N7, N31, N52, and N69 (104); in ZIKV, it occurs at N69, (105); in JEV, at N15 and T17 (95, 96); and in WNV at N15 (106). The loss of prM N-glycosylation in ZIKV resulted in protein aggregation and activation of the ER stress response, negatively impacting the ZIKV infectious cycle (105). Similarly, glycosylation-null JEV mutants at E (N154) and prM (N15 and T17) residues demonstrated poor viral particle formation and secretion, indicating the importance of glycosylation motifs in proper folding and viral assembly (95, 96). Removal of prM and E glycosylation in WNV resulted in the production of modestly infectious particles (106). Overall, the glycosylation of both E and prM proteins in orthoflaviviruses plays an essential role in viral stability, immune evasion, and pathogenicity, making it a critical factor in both the immune response and vaccine development.

### 2.5 Host genetics

Host genetic variation, particularly in immune-related genes, significantly modulates the immune response through immune imprinting (107–109). One of the most influential regulators of adaptive immunity is the MHC encoded by the highly polymorphic human leukocyte antigen (HLA) gene locus. The polymorphism of MHC molecules results in the variability of peptides presented to T cells, as different MHC alleles possess unique peptide-binding grooves. This genetic variation can influence the efficiency and specificity of immune responses, including those generated by B cells. Specific alleles have been associated with both protective and increased susceptibility to severe disease outcomes, with distinct associations observed across various populations. Variations in classical HLA class I (such as HLA-A and HLA-B) and HLA class II (such as HLA-DR, HLA-DQ, and HLA-DP) molecules determine resistance, susceptibility, and severity of the infected host to orthoflaviviruses (110, 111). In the Vietnamese population, HLA class I alleles, such as HLA-A*33, have been linked to a lower risk of severe dengue hemorrhagic fever (DHF), while allele HLA-A*24 is associated with increased risk of DHF, especially during DENV-2 infections (112). HLA-DRB1*0901 has shown protective effects against developing severe dengue shock syndrome (DSS) (113). In a larger ethnic Thai cohort, HLA-A*0207, HLA-B*51, and HLA-B*52 predisposed individuals to DF or DHF (110). In contrast, HLA-A*0203 was linked to less severe dengue fever (DF), while HLA-B*44 and B*62 were protective against severe diseases in secondary infections. These associations were partly explained by the strong peptide-binding affinities of specific alleles, which facilitated T cell activation and enhanced protection. Among HLA class II alleles, HLA-DRB1*15:01 has been associated with increased interferon-gamma secretion, suggesting a role in enhancing immune responses (114). In contrast, HLA-DRB1*03 and HLA-DRB1*09 were linked with reduced risk of severe dengue, highlighting its potential protective role (115). Finally, robust CD4+ T cell responses associated with HLA-DRB1*0401, DRB1*0701 are linked to resistance, while weaker responses related to DRB1*08:02 increased susceptibility to severe dengue (116). These findings suggest that HLA alleles and their variations could inform disease risk or vaccine strategies, and are critical for immune imprinting.

Recent studies involving monozygotic twins have shown that genetic differences in MHC molecules can shape immune imprinting that is not solely determined by environmental exposures. Despite dissimilar memory responses to past exposures, both twins developed similar subtype preferences following influenza vaccination (117), underscoring the influence of genetic factors on immune imprinting.

## 3 Future directions

A review of *in vitro*, *in vivo*, or *ex vivo* human studies suggests that cross-reactive immunity generated by prior exposure to mosquito-borne orthoflaviviruses can influence the outcome of subsequent heterologous orthoflavivirus infections (118). However, many of these studies failed to track outcomes after sequential infections, particularly in non-diseased populations, leading to gaps in our understanding of the long-term dynamics of immune imprinting. Although few studies have explored cross-protection after repeated orthoflavivirus exposures, some evidence suggests that exposure to at least two heterologous orthoflaviviruses can protect against a third heterologous orthoflavivirus. For example, tertiary infections with different DENV serotypes, distinct from those of primary and secondary infections, often result in subclinical outcomes, indicating that prior infections can prime immune responses and confer protective immunity (4). Conversely, a tertiary dengue infection following prior ZIKV exposure can worsen disease severity, a pattern that is serotype-dependent (44). In contrast, protection is observed when the order of infection is reversed (i.e., ZIKV followed by DENV) (44). Furthermore, our recent findings of broadly neutralizing antibodies in DENV-recovered individuals with prior JEV exposure may explain the low incidence of ZIKV infections in Asia and provide a consistent framework for why West Nile virus (WNV) cases in South America are not prevalent (25, 119).

One major challenge in investigating immune imprinting through repeated orthoflavivirus exposures is the difficulty in recruiting recovered individuals with distinct exposure histories for longitudinal studies, particularly for collecting peripheral blood mononuclear cells (PBMCs) or secondary lymphoid tissues. To overcome this, we recommend establishing diverse longitudinal cohorts based on different infection and vaccination histories. Future work should integrate diverse approaches, combining epidemiology, single-cell transcriptomics, virology, novel animal models, and structure biology to provide a more comprehensive understanding of the complex and long-term effects of immune imprinting.

### 3.1 B cell repertoire and clonal evolution analyses in repeated orthoflavivirus exposures

The antibody response after repeated exposure to different orthoflavivirus is affected by the mechanistic intricacies of immune imprinting, which is influenced by the dynamic GC reaction following repeated antigen exposure. In GCs, activated B cells undergo iterative rounds of somatic hypermutation, where only the clones expressing the highest affinity receptors to the antigen are selected for clonal expansion by receiving survival signals from Tfh cells. However, a portion of MBC clones will undergo further affinity maturation, leading to inter-clonal competition and a progressive loss of MBC clonal diversity, given that selection favors clones with the highest antigen affinity to immunodominant epitopes (120, 121). Nevertheless, GC reactions continuously recruit activated B cells concurrently, leading to the breadth of the humoral response. Up to 30% of late-stage GCs consist of clones with limited rounds of mutations and low antigen affinity, suggesting that the entry and expansion of B cells specific for non-dominant epitopes is favored, thereby contributing to the maintenance of antibody diversity (122). Future studies should focus on the balance between clonal expansion, affinity maturation, and the preservation of diversity, using lineage tracing to explore the landscape of B cell repertoire evolution and competition (123).

To dissect the cellular interactions that shape these GC dynamics during sequential heterologous flavivirus exposures, newly developed tools such as the uLIPSTIC (universal labelling immune partnerships by SorTagging intercellular contacts) (124) mouse model present exciting opportunities. Using *Staphylococcus aureus* transpeptidase sortase A (SrtA) to covalently transfer a peptide substrate containing the motif LPETG onto an amino-terminal pentaglycine (G_5_) acceptor to label interacting cells, uLIPSTIC enables labelling of transient cell-cell interactions *in vivo*. A *Rosa26^uLIPSTIC^* mouse model, expressing a high level of mSrtA, was developed to identify GC-resident T follicular helper cells based on their ability to interact with germinal center B cells. Applying this system in orthoflavivirus models could help distinguish the T helper cells provided to recalled versus *de novo* B cell clones. When combined with fate-mapping or single-cell analysis, uLIPSTIC could provide a high-resolution view of GC dynamics during heterologous orthoflavivirus infections. Overall, these approaches would enhance our understanding of immunodominance, clonal dynamics, and how these processes influence long-term protective immunity, ultimately informing vaccine strategies to broaden and strengthen humoral responses (125, 126).

### 3.2 Landscape mapping of serum-level antibody

A key challenge in flavivirus immunology is deciphering the composition and origin of serum polyclonal antibody responses following sequential infections or vaccinations. The complex interplay between MBCs and GC reactions, often involving multiple rounds of affinity maturation, shapes the circulating polyclonal antibody pools and ultimately contributes to the outcomes of OAS. Despite the importance of understanding the origin and composition of serum polyclonal antibody pools, molecular analyses of immunoglobulin genes obtained from memory or GC B cells do not directly assess the composition of antibodies in the serum, limiting our ability to resolve the functional dynamics of polyclonal responses. Recent advances are beginning to overcome this barrier. In particular, the neutralization fingerprinting analysis framework (127) deconvolutes the serum antibody specificity using panels of well-characterized type-specific or cross-reactive monoclonal antibodies. This approach has already been applied to assess responses in seronegative and seropositive individuals receiving the TAK-003 tetravalent dengue vaccine, currently in phase 3 trial, providing a scalable framework for mapping neutralization specificities. Complementing this, the molecular fate-mapping approach (49) enabled researchers to trace the origins of serum antibodies back to specific B cell cohorts. Adapting this technique to heterologous orthoflaviviruses would enable the precise dissection of sequential exposures at the serum level. These recent advances would inform the rational design of vaccines that aim to balance breadth and specificity across antigenically diverse orthoflaviviruses.

### 3.3 Fine mapping of the antigenic structure of orthoflaviviruses

The antigenic relationships of orthoflaviviruses, conceptualized by Calisher in 1989 as “antigenic mirrors,” offer a valuable framework for understanding cross-reactive immunity (3). Future research should focus on expanding our understanding of the antigenic relationships between orthoflaviviruses, particularly by integrating structural and functional analyses of cross-reactive antibodies. Additionally, orthoflaviviral anti-envelope (E) antibodies are categorized into three categories based on their specificity: group-reactive (GR), complex-reactive (CR), and type-specific (TS) (88). While current knowledge of the antigenic structure of orthoflavivirus is primarily based on a limited set of antibodies derived from DENV or ZIKV infections (128, 129), with structure data based on cryo-electron microscopy or X-ray crystallography, there is a need for more comprehensive mapping of the antigenic determinants that drive cross-reactive responses after repeated exposure to different orthoflaviviruses. Emerging techniques, such as electron microscopy-based polyclonal epitope mapping (EMPEM), have shown promise in finely delineating epitope specificities of serum antibodies in response to natural infection or vaccination (130, 131). Combining structural mapping with the analysis of the landscape of MBC repertoires from the cohort with complex exposure history will enhance our understanding of how immune imprinting shapes humoral immunity and clinical outcomes. These insights could inform strategies for designing more effective vaccines and therapies that take into account cross-reactivity and long-term dynamics of immunity.
